# Specifying content and mechanisms of change in interventions to change professionals’ practice: an illustration from the Good Goals study in occupational therapy

**DOI:** 10.1186/1748-5908-7-100

**Published:** 2012-10-18

**Authors:** Niina Kolehmainen, Jill J Francis

**Affiliations:** 1 Health Services Research Unit, University of Aberdeen, Aberdeen, UK; 2 Aberdeen Health Psychology Group, University of Aberdeen, Aberdeen, UK

**Keywords:** Complex interventions, Developing interventions, Behaviour change, Professionals’ practice, Goal setting, Occupational therapy

## Abstract

**Background:**

It is widely agreed that interventions to change professionals’ practice need to be clearly specified. This involves (1) selecting and defining the intervention techniques, (2) operationalising the techniques and deciding their delivery, and (3) formulating hypotheses about the mechanisms through which the techniques are thought to result in change. Descriptions of methods to achieve these objectives are limited. This paper reports methods and illustrates outputs from a study to meet these objectives, specifically from the Good Goals study to improve occupational therapists’ caseload management practice.

**Methods:**

(1) Behaviour change techniques were identified and selected from an existing matrix that maps techniques to determinants. An existing coding manual was used to define the techniques. (2) A team of occupational therapists generated context-relevant, acceptable modes of delivery for the techniques; these data were compared and contrasted with previously collected data, literature on caseload management, and the aims of the intervention. (3) Hypotheses about the mechanisms of change were formulated by drawing on the matrix and on theories of behaviour change.

**Results:**

(1) Eight behaviour change techniques were selected: goal specified; self-monitoring; contract; graded tasks; increasing skills (problem solving, decision making, goal setting); coping skills; rehearsal of relevant skills; social processes of encouragement, support, and pressure; demonstration by others; and feedback. (2) A range of modes of delivery were generated (*e.g.*, graded tasks’ consisting of series of clinical cases and situations that become increasingly difficult). Conditions for acceptable delivery were identified (*e.g.*, ‘self-monitoring’ was acceptable only if delivered at team level). The modes of delivery were specified as face-to-face training, task sheets, group tasks, DVDs, and team-based weekly meetings. (3) The eight techniques were hypothesized to target caseload management practice through eleven mediating variables. Three domains were hypothesized to be most likely to change: beliefs about capabilities, motivation and goals, and behavioural regulation.

**Conclusions:**

The project provides an exemplar of a systematic and reportable development of a quality-improvement intervention, with its methods likely to be applicable to other projects. A subsequent study of the intervention has provided early indication that use of systematic methods to specify interventions may help to maximize acceptability and effectiveness.

## Background

Implementation science is concerned with changing healthcare professionals’ practice. An approach that is frequently adopted to achieve this is the development and use of behaviour change interventions. There is a wide consensus that behaviour change interventions need to be clearly specified in order to have maximum likelihood of effectiveness and to be replicable [1-5]. Specifying interventions involves three objectives: (1) *select and define the intervention techniques*; (2) *operationalise the techniques and decide their delivery* (including mode, context, dosage, and frequency); and (3) *formulate hypotheses about the mechanisms* through which the techniques are thought to result in change [1,3,5-8]. Few among existing interventions to change professionals’ practice have been clearly specified [9]. One likely reason for this is that there are currently only limited exemplars and no agreement on methods for specifying interventions. In other words, there is a consensus about the standards for reporting interventions before evaluation but little information about the ways to achieve these standards. Frameworks such as the UK Medical Research Council (MRC) framework for complex interventions [1,2] and intervention mapping [10] have been very useful in guiding the methods for overall intervention development; however, they do not provide details on systematic methods for *specifying* the interventions.

Two papers, describing development of interventions to improve mental health professionals’ disclosure of dementia [11] and general practitioners’ management of upper respiratory tract infections [12], represent a significant step in methods for specifying interventions to change professionals’ practice. With regard to the three dimensions of specifying interventions, these papers describe a replicable method for selecting intervention techniques through the use of a behaviour change technique matrix [13] and labels for methods related to operationalising the techniques (“iterative process using the study team members” [11,12]) and deciding the delivery of the intervention techniques (“cognitive interviewing” [11,12]). Other subsequent publications have provided further labels for methods related to operationalising intervention techniques (*e.g.*, “designed a prototype” or “conducted usability testing”). These papers have made a valuable contribution to the field; however, there are aspects that limit their replicability. Specifically, these papers do not describe methods for defining the techniques (*e.g.*, how is it decided what ‘persuasive communication’ or ‘modeling’ consist of?), and they do not describe the content and application (*i.e.*, the procedure) of the methods (only the label) for operationalising the intervention techniques and for deciding their delivery and formulating hypotheses. Further exemplars with fuller descriptions of content and application of the methods are required to allow implementation scientists to specify interventions to the agreed standard.

The present paper builds on existing publications by replicating the previously described methods where possible and further developing and elaborating them. The aim is to provide an exemplar of systematic methods for specifying interventions to change professionals’ practice.

**Figure 1:**
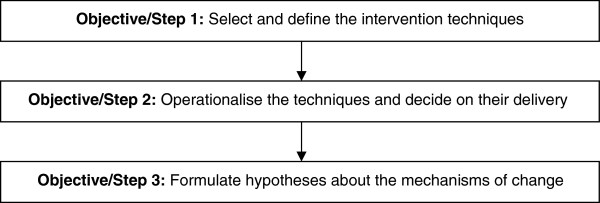
The objectives related to specifying interventions and the steps taken in the present study.

There is no established consensus for reporting studies concerning developing and specifying interventions or on the quality criteria against which the methods should be evaluated. In terms of quality criteria, as quality of randomised controlled trials is evaluated on the basis of reliability and validity of the methods used [7] (not on the trial outcomes), quality of studies focusing on developing and specifying interventions should also be evaluated on the basis of their methods (rather than, *e.g.*, the ultimate effectiveness of the intervention). For the present study, we aspired to three quality criteria: (1) the methods are replicable by others, (2) the methods systematically synthesise evidence and theory about the techniques that should be included in the intervention and about the delivery of these techniques, and (3) the application of the methods results in an intervention that is clearly specified (in terms of its techniques, their delivery, and mechanisms of change). In terms of reporting, the present paper is structured according to the three objectives related to specifying interventions (see above, and Figure 1). The following sections report the context of the study and the methods and outputs as they relate to each objective.

## Methods

### The study context and the theoretical model of the problem

The present study was part of a program of research aimed at improving efficiency and equity of occupational therapists’ caseload management. Efficiency and equity are major challenges to most health services, and professionals’ caseload management practices (*i.e.*, their behaviours related to assessment, treatment, and discharging of patients) may provide a way of addressing these [14-17]. A theoretical model of professionals’ caseload management [17] (based on synthesis of qualitative and quantitative evidence and theories of human behaviour) indicates that effective caseload management is predicted by professionals’ three behaviours: (1) identify clear and specific treatment goals, (2) agree the treatment goals with the patient, and (3) evaluate the patient’s progress towards the goals at a specified date. According to the model [17], professionals’ performance of these three ‘target behaviours’ is related to variables within seven ‘theoretical domains’ (*i.e.*, clusters of behavioural determinants [18]). These seven theoretical domains are as follows: professional role and identity, skills, beliefs about capabilities, motivation and goals, social influences, emotion, and behavioural regulation [17]. In other words, the model proposes that the frequency with which a professional, for example, agrees goals with patients is related to the professional’s belief about whether or not agreeing goals with patients is part of his or her role and responsibilities, the professional’s skills in agreeing goals with patients, and the importance that agreeing goals with patients has to the professional when also considering other priorities that he or she has. The model was used as the initial theoretical basis [2] for the present study. The present study did not require research ethics committee approval as it involved collaboration with an advisory team rather than collection of new data.

### Three steps to specifying the intervention

As noted above (in Background), specifying interventions involves three objectives: (1) select and define the intervention techniques to be delivered, (2) operationalise the techniques and decide on their delivery, and (3) formulate hypotheses about the mechanisms of change. In the present study, each of the objectives was met through a corresponding step (Figure 1). The steps mirrored the broad approach to developing complex interventions recommended by the MRC [1,2] and, more specifically, the way in which the causal modeling approach [19] has been applied in the development of interventions to change practice [11,12]. The following sections report the specific methods used to conduct each of the three steps.

### Step 1: select and define the intervention techniques to be delivered

Specifying an intervention requires selecting the techniques to be delivered (*i.e.*, the ‘active ingredients’ designed to be used to produce change in the outcomes [1]). Ideally, the techniques are chosen on the basis of explicitly theorised empirical evidence of effectiveness from previous studies in the field [19]. Empirical evidence about the effectiveness of different techniques in changing professionals’ practice is scarce [20,21], and therefore, other approaches are needed. One recommended approach is to use evidence-based theories from other fields [22,23]. Various tools have been developed to facilitate this; one of these is the matrix that consists of 35 behaviour change techniques and eleven theoretical domains of behavioural determinants [13]. The matrix has been derived, using expert consensus, from a range of theories related to professionals’ practice [13], and it indicates whether or not there is expert endorsement of a specific technique (*e.g.*, ‘graded tasks’) to be used to target specific domains (*e.g.*, ‘skills’). In the matrix, individual techniques may be proposed to target several domains (*e.g.*, the ‘graded tasks’ technique has been proposed to target the domains ‘skills’, ‘beliefs about capabilities’, and ‘motivation and goals’), and similarly, it has been proposed that some domains can be changed by using a number of techniques. The matrix can be used to systematically identify techniques for inclusion in interventions to change practice [11,12], and the matrix was therefore used in the present study.

To systematically identify relevant techniques from the matrix, the seven theoretical domains (*i.e.*, professional role and identity, skills, beliefs about capabilities, motivation and goals, social influences, emotion, and behavioural regulation) related to the target behaviours [17] were used to work through the rows of the matrix. Specifically, the behaviour change techniques that, according to the matrix, target at least two of the seven domains were identified and selected for inclusion in the intervention. All other techniques (*i.e.*, those that were not selected for inclusion) in the matrix were excluded.

The publication that reported the matrix [13] also reported a list of labels for behaviour change techniques (and brief definitions for some of them) that were extracted from publications and textbooks and elicited from researchers to generate a starting point for matrix development. In parallel with the development of the matrix, a coding manual for behaviour change interventions [24] was published. It provides explicit definitions for 26 behaviour change techniques and reports evidence of reliability of the definitions. That coding manual was used in the present study to define the selected techniques. The process for matching the techniques from the matrix with those in the coding manual are reported in Results (Step 1). The next step was to operationalise the selected techniques and decide on their delivery (see below).

### Step 2: operationalise the techniques and decide on their delivery

Operationalising the selected techniques involves deciding how the techniques will be put into practice. In the present study this was done by collaborating with an advisory team of occupational therapists. The criteria used to select the team were as follows:

▪ the team membership combines experience in clinical and professional issues and in a range of contextual factors (*e.g.*, geographical mix, client mix, organizational structure);

▪ the team members are motivated and committed to involvement in the intervention development;

▪ the team is accustomed to critical and constructive discussion; and

▪ the team is based within a manageable travel distance from the research team.

Six teams were considered; one team that met the criteria was approached and agreed to be involved. Two 60-minute meetings with the team were arranged. At the meetings, the target behaviours, the seven theoretical domains, and the intervention techniques selected (see Step 1 above) were presented to the team in the following form:)

"“The aim is to increase therapists’ [the name of the theoretical domain, e.g., ‘skills’ or ‘beliefs about capabilities’] in [the behaviour, i.e., ‘identifying goals’ or ‘agreeing goals’ or ‘evaluating progress towards goals’]. We want to do this through [the name of the behaviour change technique, e.g., ‘graded tasks’ or ‘self-monitoring’]. Can you think of ideas for how we could do this?”"

Definitions for the behaviour change techniques (see Step 1, above) and examples of the factors within the theoretical domains were also presented to the team. (The examples were based on the data underpinning the model of caseload management.) The team was encouraged to first brainstorm a range of ideas for operationalising the techniques in an uncritical manner before discussing the acceptability of these ideas. The role of the researcher was to record and prompt discussion. The team was not invited to identify additional techniques; the focus was solely on operationalising the techniques identified at Step 1. After each meeting, the team’s responses were compared against

▪ the technique definitions from Step 1;

▪ previously collected qualitative and quantitative data about occupational therapists’ caseload management [17];

▪ literature related to the topic (*i.e.*, literature about community health and social care professionals’ caseload management practice) [25]; and

▪ literature about professionals’ practice and human behaviour and the aims of the intervention.

The team’s responses that were compatible with these were accepted and included in the intervention protocol without modification. The responses that were (either in whole or in part) incompatible with these were put aside for further consideration and eventually disregarded—the implications of this are further discussed in results for Step 2. The third objective, and the final step of the study, was to formulate hypotheses about the mechanisms of change; this is described next.

### Step 3: formulate hypotheses about the mechanisms of change

Hypotheses about the mechanisms of change (*i.e.*, the relationships between the techniques identified in Step 1, the hypothesized determinants of the target behaviours, and the target behaviours themselves) were developed. This was done in two parts. First, the first author used the matrix of behaviour change techniques [12] to develop hypotheses about the theoretical domains that each technique is likely to target. Specifically, the consensus panel’s agreement that a technique could be used to target a domain was taken as sufficient evidence to hypothesize that there may be a causal relationship between the technique and any of the behavioural determinants within that domain.

Second, theories of human behaviour were used to identify any relationships that may exist between the techniques and the determinants that are not reflected in the matrix. Specifically, the first author identified three theories that cover the determinants relevant to this study and also include behaviour change techniques: Social Cognitive Theory [26], Control Theory [27], and Goal and Implementation Intentions [28]. The two authors then discussed the hypotheses presented in these theories and identified the hypotheses concerning any of the selected techniques having an impact on any determinants residing within the seven domains relevant to the target behaviours. The identified hypotheses were added to those already included on the basis of the matrix.

## Results

The following sections report the outputs that resulted from the present study. The outputs are presented in relation to each objective/step.

### Step 1: selected intervention techniques and their definitions

Ten behaviour change techniques that met the criteria were identified from the matrix (Table 1). A limitation in the matrix was noted in relation to one technique (*i.e.*, ‘feedback’); both theory and evidence strongly indicate that this technique is likely to be useful for targeting behavioural regulation, but the matrix does not indicate this. A decision was taken to include feedback; as a result, eleven techniques were identified.

**Table 1:** **Techniques identified for inclusion in the intervention**, **whether a definition was available from the coding manual**, **action taken**, **and the definitions adopted**

**Technique**	**Definition available[**[24]**]**	**Action taken**
Goal specified	Yes	Used the definition from the coding manual:
		*“A very specific definition of the behaviour, with at least one of the following specified: where, when, how, or with whom”*
Contract	Yes	Used the definition from the coding manual:
		*“Agreement so that there is a written record witnessed by another”*
Self-monitoring	Yes	Used the definition from the coding manual:
		*“Keeping a record of specified behaviour”*
Rewards	Yes	Used the definition from the coding manual:
		*“Praise, encouragement, and/or material rewards—the reward must be explicitly linked to the achievement of specified goals”*
Graded tasks	Yes	Used the definition from the coding manual:
		*“A sequence of tasks that start from easy and become increasingly difficult”*
Increasing skills (problem solving, decision making, goal setting)	No	The technique was excluded because
		▪ there was no evidence to suggest that therapists required intervention with respect to decision-making or problem-solving skills [17], and
		▪ goal-setting skills were already targeted extensively through the techniques of graded tasks and rehearsal
Coping skills	No	Technique was excluded (see results for step 2)
Rehearsal of relevant skill	Yes	Used the definition from the coding manual:
		*“Repeat the behaviour or preparatory behaviours numerous times”*
Social processes of encouragement, pressure, and support	Definitions for four similar techniques	Used the definitions for the four similar techniques (‘general encouragement’, ‘opportunities for social comparison’, ‘social support/change’, and ‘information about others’ approval’) to produce a single definition:
		*“Opportunities for mutual support, sharing, and comparison, including clarification of whether others like, approve of, or disapprove of what one is doing”*
Modeling/ demonstration by others	Yes	Used the definition from the coding manual:
		*“Showing how to perform a behaviour correctly”*
Feedback	Yes	Used the definition from the coding manual:
			*“Providing data about or commenting on a person’s action in relation to a set goal or in relation to the performance of others”*

Definitions from the coding manual [24] were available for eight of the eleven techniques (Table 1); further consideration was required for the remaining three techniques. One technique (social processes of encouragement, pressure, and support) was included in the intervention, and a definition for it was generated by combining technique definitions from the coding matrix. One technique (increasing skills: problem solving, decision making, goal setting) was dropped from the intervention at Step 1 (Table 1) and another at Step 2 (see following section) as they were not considered relevant. As such, definitions were established for all included techniques.

### Step 2: operationalised techniques and their delivery

The advisory team consisted of eight senior occupational therapists and one technical instructor. The outcomes from the meetings have been summarized in relation to each behaviour change technique in Table 2; full materials and minutes from the meetings are available from the first author. In addition to the advice related to the specific techniques, the team also recommended that the intervention should not require the service to change their paperwork (*i.e.*, the way client notes, reports, etc. are recorded) and that the intervention should include tools to support implementation in practice as opposed to training sessions only.

**Table 2:** **Summary of the clinical advisory team**’**s comments and recommendations regarding the acceptability and delivery of the specific intervention techniques**

**The technique**	**Comments about acceptability and delivery**
Goal specified AND	Having specific goals for therapists (as opposed to therapy goals for patients) is likely to range from contentious to highly unacceptable.
contract	
	Having goals for a team is likely to require persuasion, and success is likely to depend on the goals. Supportive and encouraging, rather than normative, team goals are likely to be more acceptable.
	Goals that allow measurement of progress or comparison between individual therapists are likely to be highly unacceptable.
	“Targets” are likely to be associated with “sales” and thus likely to be strongly opposed to therapists’ professional identity.
Self-monitoring AND feedback	In general, any monitoring or feedback, and especially external monitoring/feedback, about individual therapists’ practice is likely to be highly unacceptable.
	Low levels of self-monitoring might be acceptable if combined with use of social processes of encouragement and support.
Rewards	Social support and encouragement is valued very highly.
Graded tasks AND	Highly desirable, especially for the target behaviours of formulating goals and agreeing goals—for as long as the tasks were presented in a way that was relevant to practice.
Rehearsal of relevant skills	
	Could involve grading the target behaviours in terms of the other people involved (*e.g.*, whether goals are formulated with parent or with the child) and context.
Coping skills	Current method of coping with emotional aspects of practice is to draw on professional community for support; this is effective and preferable to therapists.
Social processes of encouragement and support	Emphasis should be on mutual support, positive interactions, and sharing.
	Changing practice as part of a team is likely to be more acceptable than changing practice individually.
	This technique should be included in all aspects of the intervention as far as possible and in high dose and frequency.
Social processes of pressure	It might be acceptable to establish some team norms, but these would need to be carefully negotiated if therapists’ motivation to comply with the norms is hoped to be gained.
	Explicit social pressure from colleagues or manager is likely to be highly unacceptable, and the intervention should be designed so that it cannot be used to exert pressure.
	It might be acceptable to include expectations from parents, but acceptability of this is likely to be contingent on therapists’ holding a professional norm about the importance of client-centred practice.
	Any technique that is not, or appears not to be, in line with being an autonomous practitioner is likely to be rejected.
Modeling/ demonstration of the behaviour	Examples by others, as part of the social processes of support, would be desirable.

Following a comparison and contrasting of the advisory team’s recommendations with the other sources of evidence/theory (see Methods, Step 2), the key decisions were as follows:

(i) the intervention would be delivered to a whole service (as opposed to an individual clinician) to increase the likelihood of acceptability and effectiveness [29];

(ii) the ‘social processes of encouragement and support’ technique was delivered throughout the intervention in as large and frequent amounts as possible;

(iii) emotional support and rewards were built into the ‘social processes of support and encouragement’ technique, as the team identified this as the current and preferred method of coping with emotions related to practice, and the techniques ‘coping skills’ and ‘rewards’ were dropped; and

(iv) ‘social processes of pressure’ and ‘self-monitoring’ were built into the intervention covertly as team-level self-monitoring (*i.e.*, teams within the service and therapists within the teams self-monitor actions and progress).

The main outputs from this step were three intervention components, with a list of the intervention techniques included in each component, and a manual to facilitate standardizing the delivery of the components and the techniques within them. The components, the techniques included in them, and their delivery are summarized in Table 3 (please contact the first author for the full manual). The components are two full-day training sessions, two tools for change, and team workbooks. The context of delivery for all three components is participants’ place of work (or, for component 1, a related training facility). Component 1 is delivered to the whole service by a trained facilitator; components 2 and 3 are self-administered by the teams.

**Table 3:** **Summary of the intervention components**, **related behaviour**-**change techniques**, **and summary description of the delivery of the techniques**

**Intervention component**	**Intervention techniques**	**Delivery of the techniques**^**a**^
**Training sessions**: two face-to-face, full-day events	Goal specified	A task sheet for the whole service to discuss and to agree on a caseload management goal that they will achieve in the next six months.
	Contract	
	Graded tasks	Group tasks (two to three people) starting easy and becoming increasingly difficult. The tasks focus on the target behaviours in various contexts. Component skills learnt in earlier tasks are repeated in subsequent tasks.
	Rehearsal	
	Social processes of encouragement, support, and pressure	Processes within the above group tasks.
	Modeling/demonstration by others	DVD clips from interviews with parents and other occupational therapists.
	Feedback	Facilitator’s and peer’s comments during the above group tasks.
**Tools for change**:		
1) Pre-appointment question	Graded tasks	A question to elicit goals from parents and school to make it easier for therapist to have this information.
2) Statement for appointment letters	Social processes of pressure	A statement of therapist’s commitment to formulate and agree goals and evaluate progress with patients.
**Team workbooks** to be used after the training sessions in actual context of practice	Goal specified	The goal-contract from the training sessions (above) was included as the first page of the workbooks.
	Contract	
	Self-monitoring	Team-level, paper-based tasks for teams to complete in weekly 45-min meetings. The tasks consisted of broad, open-ended questions to the teams, related to (i) their progress towards the agreed upon service-level goal and (ii) their performance of the target behaviours.
	Feedback	Peer’s comments that are guided by the workbook tasks
	Social processes of encouragement, support, and pressure	Guidance for the team on how to structure the weekly meetings. The guidance has been designed so as to activate these two techniques.
	Modeling/demonstration by others	

^a^The frequency and duration for which the techniques should be applied were also specified for each task included in the component; the full specification is available from the first author.

At the end of step 2, the intervention was titled Good Goals. In its current form, Good Goals is designed to be delivered by a trained facilitator with sufficient experience of the National Health Services (child health) context and the relevant competencies for delivering the behaviour change techniques included in the intervention (but not an expert in clinical practice and/or in science of behaviour change).

### Step 3: hypotheses about the mechanisms of change

A total of 20 hypotheses were formed; a summary of these is presented in Table 4. Eleven of the hypotheses were formed solely on the basis of the matrix, two were formed solely on the basis of a theory, and seven were formed on the basis of both the matrix and the theory.

**Table 4:** **Behaviour**-**change techniques included in the intervention and the theoretical domains they were hypothesized to target**

**Behaviour-change techniques**	**Theoretical domains**						
	**Role and identity**	**Skills**	**Beliefs about capabilities**	**Motivation and goals**	**Social influences**	**Emotion**	**Behavioural regulation**
Goal specified	*—*	*—*	*—*	M	*—*	*—*	M+T
Self-monitoring	*—*	M	M+T	*—*	*—*	*—*	T
Contract	*—*	*—*	*—*	M	*—*	*—*	M+T
Graded tasks	*—*	M	M+T	M	*—*	*—*	*—*
Rehearsal of relevant skills	*—*	M	M+T	*—*	*—*	*—*	*—*
Social processes of encouragement, support, and pressure	M	*—*	M+T	M	M	*—*	*—*
Modeling/ demonstration by others	*—*	*—*	M+T	*—*	M	*—*	*—*
Feedback	*—*	*—*	M				T

M = technique is hypothesized to target the domain on the basis of the matrix [13]; T = technique is hypothesized to target a variable within the domain on the basis of theory [26-28,30-33]; M+T = technique is hypothesized to target the domain, or a variable within the domain, on the basis of both the matrix and theory; *—* = technique is hypothesized not to have an effect on factors within the domain.

All included techniques were hypothesized to work through at least two domains. The domains that were hypothesized to be mostly targeted were beliefs about capabilities, motivation and goals, and behavioural regulation. Six of the seven domains (all except emotion) were hypothesized to be targeted (please see Results, Step 2 for operationalisation of the techniques to support coping with emotions related to practice).

## Discussion

The present paper provided an exemplar of methods and outputs from a study to specify an intervention to change healthcare professionals’ practice (specifically, occupational therapists’ caseload management). The specific target behaviours were (1) identify clear and specific treatment goals, (2) agree the treatment goals with the patient, and (3) evaluate the patient’s progress towards the goals at a specified date. The methods included (i) use of existing theory and tools to select and define intervention techniques, (ii) systematic procedures for receiving and considering advice about acceptability and delivery from an advisory group (a clinical team of occupational therapists), and (iii) development of clear hypotheses about the mechanisms through which the intervention techniques (and thus the intervention as a whole) are thought to result in change in the target behaviours.

There are a number of methodological limitations. Despite the effort to use systematic and replicable processes, it is uncertain that operationalising the intervention techniques (step 2) with a different advisory panel would result in an exact replication of the intervention as designed. For example, a different advisory team might have had different views about the acceptability of the techniques or might have suggested different tasks for operationalising the graded tasks. However, little has been published about methods for operationalising intervention techniques to date, and, despite its limitations, the present study contributes to the ongoing research [11,12,34,35] in intervention design. Also, the effects of the selected intervention techniques on the behavioural determinants were not empirically explored [12,36]. However, the clear specification of the techniques and their delivery, as well as their proposed relationships to the outcomes, will facilitate this investigation in a future effectiveness study [1,2].

The methods described in this exemplar project could be used to design and specify interventions for other implementation problems. For example, persistent implementation challenges related to professionals’ practice (*e.g.*, use of research evidence [37] and use of standardized outcome measures [38]) have been identified to relate, at least in part, to factors such as confidence, skills, and knowledge that are modifiable by behaviour change techniques. However, for application in other topics, the methods presented here have a prerequisite: there needs to be an explicit theory of the outcome being targeted. In the present project, the authors’ ability to carry out the work relied on a previously developed theoretical model of professionals’ caseload management [17]. The model provided information about the hypothesized determinants of the professionals’ practice and, through this, directly guided the selection of the intervention techniques (*i.e.*, the ‘active ingredients’ designed to achieve change in the outcome). It was only after these had been selected that it was possible to operationalise the delivery of the techniques with the clinical team (*i.e.*, to specify the mode of delivery for the techniques). Thus, it would be difficult to apply the methods presented here without first developing a theoretical model of the professionals’ action(s) that one aimed to change.

One of the challenges for the project described in this paper was identification of the quality standards against which it should be evaluated. As the methods in developing and specifying complex interventions to change practice progress, it may be that reporting standards for *development* of interventions (as opposed to just the interventions themselves) can be agreed upon. Over time, this has the potential to facilitate a cumulative science and optimise the use of resources. As intervention development becomes more systematic and transparent, it will be easier to ensure that the quality of the process used to develop individual interventions is such as to warrant their (often expensive) large-scale evaluation.

### Next steps

Good Goals is the first community caseload management intervention that has been systematically designed using theory and previous evidence [25]. Results from a recently completed study that investigated the use of Good Goals in practice [39] indicate that the issues considered during the intervention specification correspond well with the issues related to its actual use in practice. The decisions made during the intervention specification also appeared to have facilitated the acceptability of the intervention, and the change in clinicians’ performance of the target behaviours was found to be considerably larger than typically seen in studies of professionals’ practice. These findings provide some early indication that specifying interventions by using systematic methods may be advantageous.

## Competing interests

The authors declare that they have no competing interests.

## Authors’ contributions

NK, with advice from JJF, designed the study and developed the study protocol. NK and JJF selected the intervention techniques; NK coordinated and conducted the clinical advisory team meetings and analyzed the data from these. NK combined the techniques into an intervention and prepared the intervention manual. JJF commented on the manual. JJF provided advice and expertise throughout the study. NK prepared the first draft of the manuscript; JJF provided substantial intellectual contribution on all drafts. Both authors approved the final version of the manuscript and take responsibility for its content.

## Declaration of funding

The study was funded by the Chief Scientist Office of the Scottish Government Health Directorates (ref: CZF/1/38). The views expressed in this paper are those of the authors. The funder was not involved in the conduct of the study or preparation of the manuscript.
